# Post-stroke depression: Prevalence and relationship with disability in chronic stroke survivors

**DOI:** 10.4103/0972-2327.64643

**Published:** 2010

**Authors:** Abhishek Srivastava, Arun B. Taly, Anupam Gupta, Thyloth Murali

**Affiliations:** Center for Physical Medicine & Rehabilitation, Kokilaben Dhirubhai Ambani Hospital and Medical Research Institute, Bangalore-560029, India; 1 Department of Neurology, National Institute of Mental Health and Neuro Sciences (NIMHANS), Bangalore-560029, India; 2 Psychiatric & Neurological Rehabilitation, National Institute of Mental Health and Neuro Sciences (NIMHANS), Bangalore-560029, India

**Keywords:** Cerebrovascular accident, rehabilitation, quality of life

## Abstract

**Objectives:**

To evaluate (1) the prevalence of operationally defined depressive disorder (ICD-10) in chronic stroke subjects and (2) the relationship of post-stroke depression (PSD) with disability.

**Design:**

Cross-sectional, descriptive study.

**Setting:**

Neurological rehabilitation unit of a tertiary care university research center.

**Materials and Methods:**

Participants were those with first episode of supratentorial stroke of more than 3 months' duration with impaired balance and gait who had been referred for rehabilitation. Data were collected on demographic data, stroke data (side and type of lesion and post-stroke duration), cognition (mini mental state examination), depressive ideation (Hamilton Depression Rating Scale - HRDS), impairment (Scandinavian Stroke Scale), balance (Berg Balance Scale), ambulatory status (Functional Ambulation Category), walking ability (speed), and independence in activities of daily living (Barthel Index). Statistical analysis was done using SPSS 13.0. We carried out the chi-square test for ordinal variables and the independent t test for continuous variables.

**Results:**

Fifty-one patients (M:F: 41:10) of mean age 46.06 ± 11.19 years and mean post-stroke duration of 467.33 ± 436.39 days) were included in the study. Eighteen of the 51 participants (35.29%) met the criteria for depression. Demographic variables like male gender, being married, living in a nuclear family, urban background, and higher HRDS score were significantly correlated with PSD (*P* < 0.05). Depression was related to functional disability after stroke but to a statistically insignificant level (*P* > 0.05) and was unrelated to lesion-related parameters.

**Conclusion:**

Depression occurs in one-third of chronic stroke survivors and is prevalent in subjects referred for rehabilitation. PSD is related primarily to demographic variables and only to a lesser extent to functional disability following stroke.

## Introduction

Stroke is a major public health problem. Traditionally, epidemiological stroke studies have focused on mortality and recurrence[1,2] and not on the long-term morbidity. The prevalence of disability among stroke survivors is between 24–54%.[3] The progressive decrease in stroke mortality observed in the last few decades, and the subsequent increase of survivors with residual impairments and disabilities, have been accompanied by a growing interest in the factors that could interfere with functional outcome and quality of life.[4] Depression is considered as the strongest predictor of poor quality of life among stroke survivors.[5]

Post-stroke depression (PSD) is one of the common emotional disorders afflicting stroke survivors. Previous studies have reported prevalence rates that have ranged from 18% to 61%, depending upon patient selection and criteria used.[6,7] Diagnosis of PSD is challenging; therefore, it often remains unrecognized and/or undertreated. PSD is associated with cognitive impairment, increased mortality and risk of falls, increased disability, and worse rehabilitation outcome.[8]

There is a relatively large number of studies available on PSD,[5-10] but it is surprising to note that the attention of authors has generally been focused on the epidemiological features and the impact of PSD on functional outcome, and not on the factors responsible for it. The objectives of this study are to determine the frequency of PSD in chronic stroke survivors and to examine its relationship with disability.

## Materials and Methods

### Sample

This study was conducted in the neurological rehabilitation unit of a tertiary research center. Stroke patients who had completed initial hospitalization at peripheral hospitals and were attending rehabilitation program for achieving functional independence were screened for enrolment into this study over a 1-year period. Patients with a first-ever supratentorial stroke of more than 3 months' post-stroke duration, within the age range of 16–65 years, able to follow three-step commands, having impaired balance and gait but with ability to walk with or without support were included in the study. Patients with recurrent strokes, receptive aphasia, significant cognitive deficits affecting participation, and those with past history of depression were excluded from the study. The Institute Ethics Committee approved the protocol and written informed consent from all participants was taken prior to data collection.

### Design

A cross-sectional, descriptive study design was used. The rehabilitation physician managing the patient did the screening of the patients for the study. The patients fulfilling the inclusion criteria were evaluated on all outcome variables and then referred to the in-house psychiatrist for diagnosis of the operationally defined depressive disorder (ICD-10).[11] Sociodemographic and clinical information was obtained in a pre-designed format by a single evaluator.

### Outcome variables

Demographic data (age, gender, marital and residential status, and type of family), stroke data (side and type of lesion and post-stroke duration), cognition (Mini Mental State Examination),[12] depressive ideation (Hamilton Depression Rating Scale), impairments (Scandinavian Stroke Scale), balance (Berg Balance Scale), ambulatory status (functional ambulation category), walking ability (speed), and independence in activities of daily living (ADL - Barthel Index) were noted, and their relationship with PSD was evaluated.

### Statistical analysis

Continuous variables were expressed as mean ± standard deviation and were evaluated by the Student's t test. Similarly, categorical variables were expressed as percentage of the total and were evaluated by the chi-square test. The two-tailed probability value was set at *P* < 0.05 for statistical significance.

## Hamilton Rating Scale for Depression

Depressive ideation was assessed on the 24-item Hamilton Rating Scale for Depression (HRDS). It consists of queries on mood, sleep, somatic symptoms, anxiety, and engagement in pleasure activities on graded score of 0–4. This has been validated for assessment of depression in stroke survivors.[13]

## Scandinavian Stroke Scale

Impairment after stroke was assessed on the Scandinavian Stroke Scale (SSS). It consists of eight parameters, including consciousness, cognitive deficits, motor power, and walking ability, on a graded score of 0–12. The maximum score is 58; higher scores indicate better outcome.[14]

## Bergman Balance Scale

It evaluates 14 sitting and standing activities, each on a 5-point scale. The maximum score is 56. Higher scores indicate better balance. It has been tested on patients with stroke and has shown good inter-rater and intra-rater reliability of 0.98 and 0.99, respectively.[15]

## Functional Ambulation Category

It distinguishes six levels of support required during gait without taking into consideration any aid used. It is based on a walking distance of 15 meters. This has been validated for classifying qualitative walking handicap after stroke.[16] The test is performed with a cane but without orthosis. Levels are defined as follows:

- Level 0: the subject cannot walk at all or requires the help of two or more people
- Level I: the subject needs continuous support from one person who helps to carry the patient's weight and helps with balance
- Level II: the subject is dependent on the continuous or intermittent support of one person to help with balance and coordination
- Level III: the subject needs only verbal supervision
- Level IV: the subject requires help for stairs and uneven surfaces
- Level V: the subject can walk independently anywhere

### Walking speed

Walking speed was measured in meters per second as the subject walked across a 10-meter walkway. Subjects were allowed to use the walking aids they required and necessary assistance to compensate for any loss of balance was provided. This method has been validated and is widely used for quantitative classification of walking handicap following stroke.[17]

## Barthel Index

Independence in ADL was assessed with the Barthel Index. It can be subdivided to measure discrete functions of self-care and mobility, with an overall score of 100. This index has been widely used and for stroke subjects and has high reliability and validity and moderate responsiveness to changes in functional ability over time.[18]

## Results

**Figure 1:**
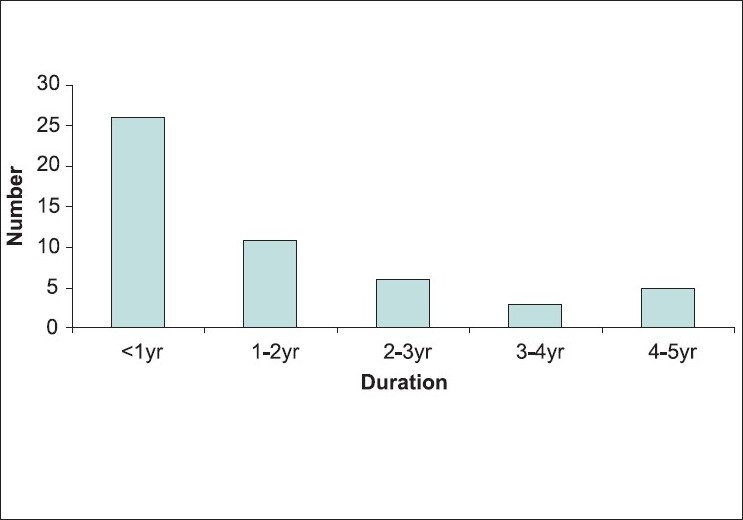
Post-stroke duration (years) of the stroke population

**Figure 2:**
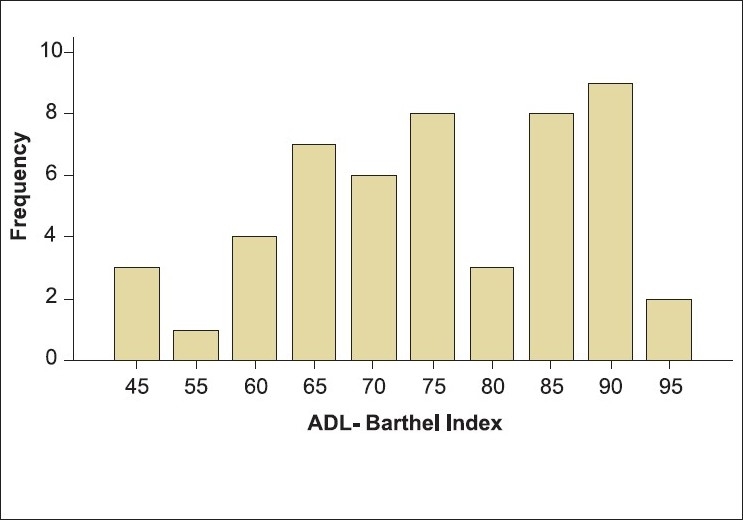
Independence in activities of daily living (Barthel Index scores) of the study population

One hundred and eighty-five stroke patients were screened during the study period and 73 of them met the inclusion criteria. Of those fulfilling the inclusion criteria, 51 patients gave informed consent and were included in the study. The clinical and demographic profile of these patients is given in Table 1. The mean age of the study population was 46.06 ± 11.19 years, mean post-stroke duration was 467.33 ± 436.39 days [Figure 1], and mean Barthel Index (BI) score was 74.33 ± 13.38 [Figure 2].

**Table 1:** Clinical and demographic profile of the study population (*n* = 51)

Variable	Value
Age (Years)	24-65 (46.06 ± 11.19)
Gender (M:F)	41:10
Marital status (UM:M:S)#	19:31:1
Type of family (N:EN:J)*	35:11:5
Residential status (R:SU:U)+	12:10:29
Type of lesion (Infarction: hemorrhage)	42:9
Side of lesion (right: left)	27:24
Post-stroke duration (days)	93-1785 (467.33 ± 436.39)
Cognitive status (Mini mental status exam)	23-30 (26.67 ± 2.35)
Depressive ideation (Hamilton scale)	1-19 (6.82 ± 3.84)
Impairment (Scandinavian stroke scale)	22-52 (40.82 ± 6.90)
Balance (Berg balance scale)	9-51 (35.75 ± 11.55)
Ambulatory status (Functional ambulation category)	II-16, III-12, IV-23
Walking ability (speed m/s)	0.10-1.02 (0.38 ± 0.26)
Activities of daily living (Barthel index)	45-95 (74.33 ± 13.38)

# UM: M: S - unmarried: married: separated);

* N: EN: J - nuclear: extended nuclear: joint);

+ R: SU: U - rural: semi-urban: urban).

Eighteen patients (35.29%) were diagnosed to have operationally defined depressive disorder as per ICD-10 criteria. Surprisingly, none of these patients were on any specific treatment for depression. When patients who were depressed (*n* = 18) were compared to those who were not depressed (*n* = 33), it was noted that depressed patients were mostly male (*P* < 0.05), married (*P* < 0.03), living in nuclear families (*P* < 0.03), from urban backgrounds (*P* < 0.03), and had higher scores on HRDS (*P* < 0.003).

Depression was not related to parameters like age, type and side of lesion, and post-stroke duration. However, when prevalence was calculated on yearly basis, there was significant reduction in prevalence in those with stroke of more than 2 years' duration [Table 2].

**Table 2:** Relationship of post-stroke duration with depression

Post-stroke duration	Number of patients	Presence of depression
< 1 year	26	12 (21.66)
1-2 years	11	4 (36.36)
> 2 years	14	2 (14.8)

Figures in parentheses are in percentage

Depression was related to functional disability, i.e., cognition, balance, ambulatory status, walking ability, and independence in ADL but the relationship did not reach statistically significance (*P* < 0.05). However, when evaluated as per different grades of independence in ADL (BI score), PSD showed high prevalence in moderately (BI score < 80) to severely (BI score < 60) dependent patients as compared to those who had mild dependency (BI score > 80) [Table 3].

**Table 3:** Relationship of functional disability (Barthel index scores) with PSD

Barthel index scores	Number of patients	Presence of depression
< 60	9	3 (33.3)
61-80	21	10 (47.6)
> 80	21	5 (23.8)

Figures in parentheses are in percentage

## Discussion

At present, despite the abundant available literature, it is still difficult to define the true prevalence rate of PSD. This variability between studies arises not only from the methodological problems of the investigations but also from the complexity in recognizing, assessing, and diagnosing depression.[8] While most of the studies have based their diagnosis on different rating scales, others have used a structured interview format and the diagnostic standards defined by International Classification of Diseases or the Diagnostic and Statistical Manual of Mental Disorders.[8] It is important to recognize that rating scales were designed to measure depression severity in primary depressive illness rather than to diagnose depression itself, especially depression occurring as a comorbidity. The correct attribution of somatic symptoms to either PSD or stroke is a very relevant issue, because such symptoms may affect rating scales. This may be compounded by the inadequacies of physicians who do not have proper psychiatric training.[19] The present study was designed taking into account all these problems. The diagnosis of PSD in this study was done by a psychiatrist on operationally defined norms (ICD-10). The Hamilton Rating Scale for Depression was used only as an adjunct and it correlated well with the diagnosis of PSD.

A meta-analysis has estimated the pooled frequency of PSD at 33%, even with relevant differences across studies.[20] In particular, the pooled estimate from the population-based studies was equal in the acute and medium-term phases (33%), with a slight increase to 34% in the long-term phase of recovery after stroke. Moreover, there were only slight differences in the pooled frequencies in the hospital-based (acute 36%, medium-term 32%, and long-term 34%) and rehabilitation-based studies (acute 30%, medium-term 36%, and long-term 34%) over time [Table 4].

**Table 4:** Comparison between nondepressed and depressed patient population

Variable	Nondepressed	Depressed	*P* value
			
	(*n* = 33)	(*n* = 18)	
Age (Years)	47.76 ± 11.93	43.17 ± 9.47	0.26
Gender (M:F)	24:9	17:1	0.05
Marital status (UM:M:S)#	14:18:1	5:13:0	0.03
Type of family (N:EN:J)*	23:7:3	12:4:2	0.03
Residential status (R:SU:U)+	7:9:17	5:1:12	0.03
Type of lesion (Infarction: hemorrhage)	7:6	15:3	1.00
Side of lesion (right: left)	17:16	10:8	1.00
Post-stroke duration (days)	529.48 ± 497.68	354.61 ± 270.25	0.17
Cognitive status (Mini mental status exam)	26.97 ± 2.49	26.11 ± 1.96	0.21
Depressive ideation (Hamilton scale)	5.70 ± 2.90	9.35 ± 4.42	0.003
Impairment (Scandinavian stroke scale)	41.73 ± 6.99	39.89 ± 6.39	0.45
Balance (Berg balance scale)	36.06 ± 11.69	34.61 ± 11.13	0.79
Ambulation (functional ambulation category) II-10, III-8, IV-15	II-6, III-4, IV-8	0.92	
Walking ability (speed m/s)	0.42 ± 0.32	0.36 ± 0.22	0.47
Activities of daily living (Barthel index)	77.27 ± 12.50	70.28 ± 12.77	0.09

# UM: M: S - unmarried: married: separated;

* N: EN: J - nuclear: extended nuclear: joint;

+ R: SU: U - rural: semi-urban: urban

The prevalence of PSD in this study was 35.29%, which approximates the pooled estimates of 34–36% for medium-term to long-term rehabilitation-based studies.[21] The difference in prevalence rate as compared to other studies[7,21-23] might be due to the sample characteristics, since this study was confined to patients who were referred for rehabilitation, that too in the late phase following stroke. These were likely to be patients who were preselected on the basis of their rehabilitation potential, and in such a sample patients with mild and very severe stroke are likely to be excluded. The relatively younger mean age of 46.06 years in our study is probably reflective of this bias. The exclusion of patients with aphasia, because of the expected difficulty in evaluating depressive symptoms, was also an important confounding variable.[24]

A major concern is that none of the depressed patients in our study were on any antidepressant treatment. PSD is associated with increased disability and worse rehabilitation outcome[9,10] and therefore the need to routinely screen chronic stroke survivors for depression and institute appropriate treatment should be emphasized. Absence of PSD in young adults is a significant predictor of the ability to return to work. Improvement of depressive symptoms has been associated with better functional recovery.[25]

Demographic variables are important determinants of PSD.[21-23] Similarly, in our study, the most important determinants of depression were demographic variables like male gender, marital status, and living situation. This reflects the sociodemographic profile of a developing country, where there is lower per capita expenditure on health and few, if any, benefits provided to the disabled population. The majority of the earlier studies[21-23] had reported female gender as being an important risk factor for PSD. However, in contrast, male gender was an important determinant in our study. This might be due to the low proportion of females in our study population.

PSD in this study was not related to stroke lesion–related parameters like age, type and side of lesion, and post-stroke duration. This is in concordance with the majority of earlier studies.[21-23] However, one study each had reported site of the lesion,[26] younger age,[21] and older age[27] as being among the important risk factors for PSD. Although, post-stroke duration was not significantly correlated to PSD in our study, when yearly prevalence was calculated, there was significant reduction in the prevalence of PSD after 2 years. This is because longer post-stroke duration does have a positive effect on emotional responses to disability.[7]

Correlation between depression and impairment in ADL peaked at 6 months and thereafter fell, but it remained significant at 1 and 2 years post stroke.[28] The persisting significant association of impairment in ADL with depression may reflect the effect of severe depression in sustaining—and possibly even retarding recovery from—physical impairment.

Depression was related to functional disability in our study, as patients with PSD have lower mean scores for all functional parameters (cognition, balance, ambulatory status, walking ability, and independence in ADL) as compared to those without depression; however, this did not reach statistically significant levels (*P* < 0.05). This is one among few studies where multiple dimensions of functional disability, such as post-stroke impairment, cognitive status, balance, walking ability, and independence in ADL are studied together.[29,30] One earlier study reported that PSD was unrelated to functional disability.[22] However, the majority of the previous studies have reported a significant relationship between disability and PSD.[23,27,28] This difference between our study and most of the earlier studies is due to two major reasons: 1) Our study population was less disabled due to a preselection bias, as patients with greater rehabilitation potential were included, and 2) the longer post-stroke duration allowed more time for the patients to come to terms with their disability.[7,28]

This study has several limitations because of the nature of the study population: for example, the small sample size, unequal gender distribution, exclusion of patients with aphasia, and longer post-stroke duration. The major limitation was the preselected nature of the study population, with only those with better rehabilitation potential being included; patients with mild or severe strokes are not likely to have been included, and the study population is therefore not representative of the general stroke population. The sample in this study was screened by the rehabilitation physicians providing care, but the diagnosis of PSD was made by a psychiatrist and that too on operationally defined guidelines; this emphasizes the need for multidisciplinary teams in rehabilitation. This is among very few studies that have evaluated the relationship of demographic factors, lesion–related parameters, and multiple functional disability parameters simultaneously in chronic stroke survivors. Such an approach aids better understanding of the origin and effects of depression following stroke. Knowledge of PSD and its relationship with disability may be of use during the planning, provision, and allocation of health services for the care of stroke survivors, especially in this era of health care cost management.

## Conclusion

The results of this study indicate that depression is underrecognized following stroke. The prevalence of PSD is high, with about one-third of stroke survivors suffering from it. Demographic variables are important determinants of PSD. Depression is related to functional disability following stroke but the relationship does not reach statistical significance in our study. The association of disability with PSD may reflect its effect on sustaining—and possibly retarding recovery from—physical impairment. We suggest that chronic stroke survivors be routinely screened for depression, given its high prevalence and its negative impact on further recovery and quality of life.

## References

[1] Kappelle LJ, Adams HP, Heffner ML, Torner JC, Gomez F, Biller J (1994). Prognosis of young adults with ischemic stroke. A long-term follow-up study assessing recurrent vascular events and functional outcome in the Iowa Registry of Stroke in Young Adults. Stroke.

[2] Lai SM, Alter M, Friday G, Sobel E (1995). Prognosis for survival after an initial stroke. Stroke.

[3] Sacco RL (1997). Risk factors, outcomes, and stroke subtypes for ischemic stroke. Neurology.

[4] Sarti C, Rastenyte D, Cepaitis Z, Tuomilehto J (2000). International trends in mortality from stroke, 1968 to 1994. Stroke.

[5] Kim P, Warren S, Madill H, Hadley M (1999). Quality of life of stroke survivors. Qual Life Res.

[6] House A (1987). Mood disorders in the first year after stroke. Nurs Times.

[7] Kong KH, Yang SY (2006). Health-related quality of life among chronic stroke survivors attending a rehabilitation clinic. Singapore Med J.

[8] Paolucci S (2008). Epidemiology and treatment of post-stroke depression. Neuropsychiatr Dis Treat.

[9] Paolucci S, Antonucci G, Pratesi L, Traballesi M, Grasso MG, Lubich S (1999). Poststroke depression and its role in rehabilitation of inpatients. Arch Phys Med Rehabil.

[10] Gillen R, Tennen H, McKee TE, Gernert-Dott P, Affleck G (2001). Depressive symptoms and history of depression predict rehabilitation efficiency in stroke patients. Arch Phys Med Rehabil.

[11] International Classification of Diseases 10 (ICD 10) for Mental and Behavioral Disorders – Clinical Descriptions and Diagnostic Guidelines. http://www.who.int/classifications/icd/en/.

[12] Folstein MF, Folstein SE, McHugh PR (1975). "Mini-mental state". A practical method for grading the cognitive state of patients for the clinician. J Psychiatr Res.

[13] Hamilton M (1960). A rating scale for depression. J Neurol Neurosurg Psychiatry.

[14] (1985). Multicenter trial of hemodilution in ischemic stroke--background and study protocol. Scandinavian Stroke Study Group. Stroke.

[15] Berg KO, Maki BE, Williams JI, Holliday PJ, Wood-Dauphinee SL (1992). Clinical and laboratory measures of postural balance in an elderly population. Arch Phys Med Rehabil.

[16] Perry J, Garrett M, Gronley JK, Mulroy SJ (1995). Classification of walking handicap in the stroke population. Stroke.

[17] Wade DT, Wood VA, Heller A, Maggs J, Langton Hewer R (1987). Walking after stroke. Measurement and recovery over the first 3 months. Scand J Rehabil Med.

[18] Mahoney FI, Barthel DW (1965). Functional evaluation: the barthel index. Md State Med J.

[19] Schubert DS, Taylor C, Lee S, Mentari A, Tamaklo W (1992). Detection of depression in the stroke patient. Psychosomatics.

[20] Hackett ML, Yapa C, Parag V, Anderson CS (2005). Frequency of depression after stroke: a systematic review of observational studies. Stroke.

[21] Hayee MA, Akhtar N, Haque A, Rabbani MG (2001). Depression after stroke-analysis of 297 stroke patients. Bangladesh Med Res Counc Bull.

[22] Cassidy E, O'Connor R, O'Keane V (2004). Prevalence of post-stroke depression in an Irish sample and its relationship with disability and outcome following inpatient rehabilitation. Disabil Rehabil.

[23] Paolucci S, Gandolfo C, Provinciali L, Torta R, Toso V; DESTRO Study Group (2006). The Italian multicenter observational study on post-stroke depression (DESTRO). J Neurol.

[24] Carson AJ, MacHale S, Allen K, Lawrie SM, Dennis M, House A (2000). Depression after stroke and lesion location: a systematic review. Lancet.

[25] Chemerinski E, Robinson RG, Kosier JT (2001). Improved recovery in activities of daily living associated with remission of poststroke depression. Stroke.

[26] Robinson RG, Starr LB, Kubos KL, Price TR (1983). A two-year longitudinal study of post-stroke mood disorders: findings during the initial evaluation. Stroke.

[27] Glodzik-Sobańska L, Slowik A, Borratyńska A, Szczudlik A (2003). Depressive symptoms following ischemic stroke. Neurol Neurochir Pol.

[28] Parikh RM, Lipsey JR, Robinson RG, Price TR (1987). Two-year longitudinal study of post-stroke mood disorders: dynamic changes in correlates of depression at one and two years. Stroke.

[29] Carod-Artal J, Egido JA, González JL, Varela de Seijas E (2000). Quality of life among stroke survivors evaluated 1 year after stroke: experience of a stroke unit. Stroke.

[30] Nagaraja D, Taly AB, Chatterjee S (1997). Post stroke mood disorders. J Assoc Physicians India.

